# Supplementary material for: New Genomic Resources for an Invasive Mite Pest Expand the Known Diversity of Pesticide Resistance Mechanisms and Guide Development of KASP Diagnostic Markers
Source: Evol Appl. 2026 Nov 2;19(11):e70344. doi: 10.1111/eva.70344 (PMC13728459; doi:10.1111/eva.70344)
Supplement: Supplementary file 1 [file EVA-19-e70344-s003.docx]

**Figure S1.** Scree plot for a PCA of nuclear SNP allele frequencies in *Halotydeus destructor*. The *x*-axis represents the first 15 PC axes, and the *y*-axis represents the percentage of explained variance. Note the elbowing at PC3, suggesting that PC1 to PC2 explain most of the population genetic structure.

**Figure S2.** Isolation-by-distance between *Halotydeus destructor* population pairs within Australia. Geographic distance is on the *x*-axis, measured in kilometers. Genetic differentiation is on the *y*-axis, measured as *F*_ST_. Points represent population pairs, coloured by region (west or east) (see legend). Panels separate population pairs from different years.

**Figure S3.** Associations between non-synonymous mutations in the *ace* gene and organophosphate resistance in *Halotydeus destructor*. The *x*-axis represents the allele frequency of each mutation, and the *y*-axis represents the proportional survival of mites following exposure to a discriminating concentration of chlorpyrifos and omethoate. Points represent mite populations, coloured by Australian state (see legend). The dashed lines indicate an expected 1:1 relationship. Panels contain data for combinations of mutation (*Torpedo californica* numbering first, *H. destructor* numbering second, separated by a bar; ‘n/a’ indicates no equivalent position in *T. californica*) and pesticide. See also Supplementary Figure S5 for associations between known target-site mutations G119S and A201S with the mutation V560I: V560I appears to have an association with chlorpyrifos, but is not a known target-site mutation.

**Figure S4.** Associations between non-synonymous mutations in the *para* gene and pyrethroid resistance in *Halotydeus destructor*. The *x*-axis represents the allele frequency of each mutation, and the *y*-axis represents the proportional survival of mites following exposure to a discriminating dose of bifenthrin. Points represent mite populations, coloured by Australian state (see legend). The dashed lines indicate an expected 1:1 relationship. Panels contain data for each mutation (*Musca domestica* numbering first, *H. destructor* numbering second, separated by a bar).

**Figure S5.** Associations between the *ace* non-synonymous G119S mutation and the A201S and V560I mutations in *Halotydeus destructor*. The *x*- and *y*-axes represent the allele frequency for each mutation (*Torpedo californica* numbering). Points represent mite populations, coloured by Australian state (see legend). (a) G119S vs A201S. The dashed line indicates an expected 1:−1 relationship. These mutations are uncorrelated in some populations, and negatively correlated in other populations. Negative correlations suggest that in some populations, these mutations are on different haplotypes. (b) G119S vs V560I. The dashed line indicates an expected 1:1 relationship. These mutations are uncorrelated in some populations, and positively correlated in other populations. Positive correlations suggest that in some populations, these mutations are on the same haplotype. Hence, associations between V560I and resistance (as in Figure S3) may be due to its linkage to G119S.

**Figure S6.** Genomic context around *ace*-like genes in cluster A (as per Figure 7). Data support two putative paralogous genes on the same genomic sequence. The *x*-axis represents the base coordinate on the assembled chromosome (or contig). The grey horizontal bar represents the genomic sequence. The magenta bars represent the *ace*-like genes. The turquoise bars represent other genes within 10,000 bp of the *ace*-like genes. Genes *above* the grey genomic sequence are those on the positive strand, whereas those *below* the grey genomic sequence are those on the negative strand. Panels separate different chromosomes (or contigs).

**Figure S7.** Genomic context around *ace*-like genes in cluster B (as per Figure 7). Data support two putative paralogous genes on the same genomic sequence. The *x*-axis represents the base coordinate on the assembled chromosome (or contig). The grey horizontal bar represents the genomic sequence. The magenta bars represent the *ace*-like genes. The turquoise bars represent other genes within 10,000 bp of the *ace*-like genes. Genes *above* the grey genomic sequence are those on the positive strand, whereas those *below* the grey genomic sequence are those on the negative strand. Panels separate different chromosomes (or contigs).

**Figure S8.** Genomic context around *ace*-like genes in cluster C (as per Figure 7). Data support two putative paralogous genes on the same genomic sequence. The *x*-axis represents the base coordinate on the assembled chromosome (or contig). The grey horizontal bar represents the genomic sequence. The magenta bars represent the *ace*-like genes. The turquoise bars represent other genes within 10,000 bp of the *ace*-like genes. Genes *above* the grey genomic sequence are those on the positive strand, whereas those *below* the grey genomic sequence are those on the negative strand. Panels separate different chromosomes (or contigs).

**Figure S9.** Genomic context around *ace*-like genes in cluster D (as per Figure 7). Data support three putative orthologous genes artificially split on different genomic sequences. The *x*-axis represents the base coordinate on the assembled chromosome (or contig). The grey horizontal bar represents the genomic sequence. The magenta bars represent the *ace*-like genes. The turquoise bars represent other genes within 10,000 bp of the *ace*-like genes. Genes *above* the grey genomic sequence are those on the positive strand, whereas those *below* the grey genomic sequence are those on the negative strand. Panels separate different chromosomes (or contigs).

**Figure S10.** Genomic context around *ace*-like genes in cluster E (as per Figure 7). Data support two putative paralogous genes on the same genomic sequence. The *x*-axis represents the base coordinate on the assembled chromosome (or contig). The grey horizontal bar represents the genomic sequence. The magenta bars represent the *ace*-like genes. The turquoise bars represent other genes within 10,000 bp of the *ace*-like genes. Genes *above* the grey genomic sequence are those on the positive strand, whereas those *below* the grey genomic sequence are those on the negative strand. Panels separate different chromosomes (or contigs).

**Figure S11.** Genomic context around *ace*-like genes in cluster F (as per Figure 7). Data support two putative orthologous genes artificially split on different genomic sequences. The *x*-axis represents the base coordinate on the assembled chromosome (or contig). The grey horizontal bar represents the genomic sequence. The magenta bars represent the *ace*-like genes. The turquoise bars represent other genes within 10,000 bp of the *ace*-like genes. Genes *above* the grey genomic sequence are those on the positive strand, whereas those *below* the grey genomic sequence are those on the negative strand. Panels separate different chromosomes (or contigs).

**Figure S12.** Genomic context around *ace*-like genes in cluster G (as per Figure 7). Data support two putative orthologous genes artificially split on different genomic sequences. The *x*-axis represents the base coordinate on the assembled chromosome (or contig). The grey horizontal bar represents the genomic sequence. The magenta bars represent the *ace*-like genes. The turquoise bars represent other genes within 10,000 bp of the *ace*-like genes. Genes *above* the grey genomic sequence are those on the positive strand, whereas those *below* the grey genomic sequence are those on the negative strand. Panels separate different chromosomes (or contigs).

**Figure S13.** Genomic context around *ace*-like genes in cluster H (as per Figure 7). Data support two putative orthologous genes artificially split on different genomic sequences. The *x*-axis represents the base coordinate on the assembled chromosome (or contig). The grey horizontal bar represents the genomic sequence. The magenta bars represent the *ace*-like genes. The turquoise bars represent other genes within 10,000 bp of the *ace*-like genes. Genes *above* the grey genomic sequence are those on the positive strand, whereas those *below* the grey genomic sequence are those on the negative strand. Panels separate different chromosomes (or contigs).

*

**Table S1.** Non-synonymous mutations in pesticide target genes of *Halotydeus destructor*. Mutations are reported as canonical reference numbering first (*Torpedo californica* for *ace*, *Musca domestica* for *para*; and *Myzus persicae* for *nAChR*), *H. destructor* numbering second, separated with a bar. An ‘n/a’ indicates an equivalent position does not exist. SNP loci are reported as the chromosome and position. Highlighted rows indicate mutations that have been linked to target-site resistance in other arthropods. Mutation frequencies are reported as their min, max, median and mean across 190 populations.

| **Gene** | **Mutation** | **SNP** | **Min** | **Max** | **Median** | **Mean** |
| --- | --- | --- | --- | --- | --- | --- |
| *ace* | n/a \| T27A | chrom_1:5714523 | 0.000 | 0.623 | 0.049 | 0.084 |
|  | S62T \| S101T | chrom_1:5714300 | 0.000 | 0.357 | 0.000 | 0.015 |
|  | G119S \| G158S | chrom_1:5714130 | 0.000 | 0.881 | 0.000 | 0.142 |
|  | I134T \| I173T | chrom_1:5714084 | 0.000 | 0.189 | 0.000 | 0.003 |
|  | A168S \| A206S | chrom_1:5713986 | 0.000 | 0.174 | 0.000 | 0.004 |
|  | A201S \| A239S | chrom_1:5713887 | 0.000 | 0.936 | 0.000 | 0.077 |
|  | S220N \| S258N | chrom_1:5713829 | 0.000 | 0.234 | 0.000 | 0.015 |
|  | F331Y \| F368Y | chrom_1:5713499 | 0.000 | 0.908 | 0.000 | 0.021 |
|  | P494L \| P535L | chrom_1:5711809 | 0.000 | 0.774 | 0.000 | 0.010 |
|  | n/a \| H579R | chrom_1:5711617 | 0.000 | 0.155 | 0.000 | 0.006 |
|  | S554N \| S607N | chrom_1:5711533 | 0.000 | 0.314 | 0.000 | 0.009 |
|  | V560I \| V632I | chrom_1:5711459 | 0.000 | 0.873 | 0.000 | 0.051 |
| *para* | Y3H \| Y3H | chrom_3:2662236 | 0.000 | 0.333 | 0.000 | 0.010 |
|  | G5A \| G5A | chrom_3:2662243 | 0.117 | 1.000 | 0.625 | 0.632 |
|  | I249M \| I253M | chrom_3:2666033 | 0.000 | 1.000 | 0.000 | 0.106 |
|  | F309I \| F309I | chrom_3:2666199 | 0.000 | 0.596 | 0.083 | 0.108 |
|  | Y485H \| Y517H | chrom_3:2666823 | 0.000 | 0.865 | 0.000 | 0.010 |
|  | D757N \| D810N | chrom_3:2671177 | 0.000 | 0.298 | 0.000 | 0.014 |
|  | M918V \| M979V | chrom_3:2671925 | 0.000 | 0.351 | 0.000 | 0.013 |
|  | M918T \| M979T | chrom_3:2671926 | 0.000 | 0.205 | 0.000 | 0.011 |
|  | D924G \| D985G | chrom_3:2671944 | 0.000 | 0.179 | 0.000 | 0.004 |
|  | L925I \| L986I | chrom_3:2671946 | 0.000 | 0.304 | 0.000 | 0.011 |
|  | F1020Y \| F1081Y | chrom_3:2672354 | 0.000 | 0.310 | 0.000 | 0.007 |
|  | L1024F \| L1085F | chrom_3:2672367 | 0.000 | 1.000 | 0.000 | 0.240 |
|  | S1027R \| S1088R | chrom_3:2672374 | 0.000 | 0.410 | 0.000 | 0.007 |
|  | L1083V \| L1146V | chrom_3:2672802 | 0.000 | 0.212 | 0.000 | 0.002 |
|  | T1146A \| T1209A | chrom_3:2672991 | 0.000 | 0.451 | 0.000 | 0.012 |
|  | Q1157R \| Q1220R | chrom_3:2673025 | 0.000 | 0.152 | 0.000 | 0.002 |
|  | F1538I \| F1610I | chrom_3:2677135 | 0.000 | 0.409 | 0.000 | 0.005 |
|  | S2045G \| S2075G | chrom_3:2678788 | 0.000 | 0.379 | 0.000 | 0.016 |
|  | S2085R \| S2086R | chrom_3:2678823 | 0.000 | 0.222 | 0.000 | 0.024 |
| *nAChR-β1* | H2L \| H4L | chrom_3:3631418 | 0.000 | 0.353 | 0.019 | 0.045 |

**Table S2.** Analysis of deviance for models testing the association between resistance with *ace* coverage (read depth) and the frequency of the G119S and A201S mutations in *Halotydeus destructor*. *P*-values significant at a threshold of α = 0.05 are shaded pink.

| **Chemical** | **State** | **Term** | **LR Chisq** | **Df** | **Pr(>Chisq)** |
| --- | --- | --- | --- | --- | --- |
| Chlorpyrifos | WA | Read depth | 0.406 | 1 | 0.524 |
|  |  | G119S freq | 141.772 | 1 | <0.001 |
|  |  | A201S freq | 0.258 | 1 | 0.612 |
|  | SA | Read depth | 5.792 | 1 | 0.016 |
|  |  | G119S freq | 134.504 | 1 | <0.001 |
|  |  | A201S freq | 5.722 | 1 | 0.017 |
|  | VIC | Read depth | 0.024 | 1 | 0.878 |
|  |  | G119S freq | 0.134 | 1 | 0.715 |
|  |  | A201S freq | 0.171 | 1 | 0.679 |
| Omethoate | WA | Read depth | 71.819 | 1 | <0.001 |
|  |  | G119S freq | 3.626 | 1 | 0.057 |
|  |  | A201S freq | 4.296 | 1 | 0.038 |
|  | SA | Read depth | 0.068 | 1 | 0.794 |
|  |  | G119S freq | 22.768 | 1 | <0.001 |
|  |  | A201S freq | 16.093 | 1 | <0.001 |
|  | VIC | Read depth | 0.221 | 1 | 0.639 |
|  |  | G119S freq | 0.573 | 1 | 0.449 |
|  |  | A201S freq | 0.377 | 1 | 0.539 |

**Table S3.** Partial regression coefficients for models testing the association between resistance with *ace* coverage (read depth) and the frequency of the G119S and A201S mutations in *Halotydeus destructor*.

| **Chemical** | **State** | **Coeff** | **Estimate** | **SE** |
| --- | --- | --- | --- | --- |
| Chlorpyrifos | WA | Intercept | −2.58 | 0.23 |
|  |  | Read depth | −0.13 | 0.2 |
|  |  | G119S | 1.77 | 0.2 |
|  |  | A201S | −0.11 | 0.22 |
|  | SA | Intercept | −1.65 | 0.17 |
|  |  | Read depth | −0.37 | 0.15 |
|  |  | G119S | 1.99 | 0.2 |
|  |  | A201S | 0.36 | 0.15 |
|  | VIC | Intercept | −6.12 | 0.89 |
|  |  | Read depth | −0.18 | 1.17 |
|  |  | G119S | 1.15 | 2.87 |
|  |  | A201S | −1.3 | 3.36 |
| Omethoate | WA | Intercept | −3.58 | 0.33 |
|  |  | Read depth | 1.27 | 0.16 |
|  |  | G119S | 0.31 | 0.16 |
|  |  | A201S | 0.39 | 0.17 |
|  | SA | Intercept | −1.18 | 0.22 |
|  |  | Read depth | −0.06 | 0.23 |
|  |  | G119S | 1.2 | 0.26 |
|  |  | A201S | 0.73 | 0.19 |
|  | VIC | Intercept | −4.84 | 0.33 |
|  |  | Read depth | −0.22 | 0.47 |
|  |  | G119S | 0.87 | 1.15 |
|  |  | A201S | −0.7 | 1.16 |
